# Role of Rab4A in the enhancement of cancer cell invasion induced by plasma samples collected during radiotherapy in patients with triple-negative breast cancer

**DOI:** 10.1371/journal.pone.0329717

**Published:** 2026-09-15

**Authors:** Benoit Paquette, Hélène Therriault, Isabelle Gauthier, Sawyna Provencher, Jean-Marc Bourque, Sahar Naasri, Ayman Oweida, Sameh Geha, Pascale Labrecque, Jean-Luc Parent

**Affiliations:** 1 Centre for Research in Radiotherapy, Sherbrooke University Cancer Research Institute, Department of Medical Imaging and Radiation Sciences, Universite de Sherbrooke, Sherbrooke, Quebec, Canada; 2 Service of Radiation Oncology, Sherbrooke University Hospital Center, Sherbrooke, Quebec, Canada; 3 Department of Radiation Oncology, Research Centre of Centre Hospitalier de l’Universite de Montreal, Montreal, Quebec, Canada; 4 Department of Pathology, Sherbrooke University Hospital Center, Sherbrooke University Cancer Research Institute, Universite de Sherbrooke, Sherbrooke, Quebec, Canada; 5 Department of Medicine, Faculty of Medicine and Health Sciences, Universite de Sherbrooke, Sherbrooke, Quebec, Canada; 6 Sherbrooke Institute of Pharmacology, Faculty of Medicine and Health Sciences, Universite de Sherbrooke, Sherbrooke, Quebec, Canada; University of California San Diego, UNITED STATES OF AMERICA

## Abstract

The relapse rate for early-stage triple-negative breast cancer (TNBC) is significantly higher than that of other breast cancer subtypes. To reduce the relapse rate, we evaluated the relevance of targeting Rab4A to inhibit the increase in cancer cell invasion induced by radiotherapy (RT). The ability of cancer cells to invade into adjacent tissues correlates with the proteolytic activity of membrane type 1 matrix metalloproteinase (MT1-MMP) on their surface, whose turnover is regulated by Rab4A. When RAB4A was downregulated using shRNA in the TNBC cells D2A1 and MDA-MB-231, a significant reduction in the proteolytic activity of MT1-MMP and the invasion capacity of these TNBC cells were measured. Plasma samples from six early-stage TNBC patients were collected before RT and after the fourth radiation dose. Plasma samples collected during RT from three of these patients increased the invasiveness of TNBC cells, compared to plasma collected before RT. Of them, two experienced a recurrence of their cancer, while the third patient had not yet completed the 5-year follow-up. The downregulation of RAB4A inhibited the RT-induced increase in cancer cell invasion. In contrast, in the three patients who did not relapse after 5-year follow-up, plasma collected during RT did not significantly increase the invasiveness of the MDA-MB-231 cells, compared to their plasma collected before RT. The decrease in RAB4A expression did not result in a significant difference in the invasiveness of cancer cells when incubated with plasma collected before or during RT from these patients. Metastatic development was observed when D2A1 cells were preincubated before i.v. injection in Balb/c mice with plasma collected during RT from a patient who experienced early cancer relapse; this was also inhibited by downregulating RAB4A. In summary, our preliminary results generate the hypothesis that RT may increase cancer cell invasion and metastasis formation, which is associated with the Rab4A-mediated turnover of MT1-MMP on the cancer cell surface.

## Introduction

The triple negative breast cancer (TNBC) subtype is characterized by low or absent expression of estrogen, progesterone and human epidermal growth factor-2 (HER2) receptors [1]. Although radiotherapy (RT) improves survival in patients with breast cancer [2], the risk of recurrence remains higher in those with TNBC [3]. The recurrence pattern is also different, occurring in about 30% of cases, which are detected as early as 6 months after treatment completion with a risk peaking at around three years [3–5].

Cancer cells can leave the primary tumor and infiltrate the breast [6]. In order to treat them, the whole breast is irradiated as well as certain locoregional lymph nodes [7]. A total dose of 26–50 Gy is delivered in 5–25 fractions [8]. Therefore, the first fractions of radiation are not lethal to a large proportion of cancer cells [9]. Additionally, the maximum dose that can be delivered is restricted by the tolerance of the surrounding healthy tissue within the irradiated volume [10]. Therefore, a lethal dose of radiation may not be delivered to all cancer cells, which could contribute to local recurrence and development of metastases [11].

Metastases detected earlier after treatment may originate from dormant micrometastases that were undetectable at diagnosis, whereas those detected later may arise from cancer cells still present after surgery [12–14]. RT can stimulate these two pathways of metastasis development. Indeed, clinical and preclinical studies suggest that RT induces changes in the tumor environment that may increase the risk of developing metastases and thus diminish the long-term effectiveness of the treatment [9,15–19].

Pre-irradiation of the mouse mammary gland followed by implantation of non-irradiated TNBC cells, or the irradiation of a TNBC tumor implanted in a mammary gland has been shown to increase cancer cell invasion in the mammary gland. This process also leads to a higher a number of circulating tumor cells, resulting in a greater number of lung metastases [20,21]. These adverse effects of RT were blocked by reducing proteolytic activity of the membrane type 1 matrix metalloproteinase (MT1-MMP) on the TNBC cells [22]. These results indicate that the higher number of circulating tumor cells and lung metastases was not significantly caused by an increase in vascular permeability but rather by a stimulation of the invasive capacity of cancer cells [23].

In both scenarios of activation of dormant metastases and of those originating from cancer cells remaining locally after treatment, the remodelling of extracellular matrix proteins, among other things, facilitate the invasion of cancer cells and the expansion of metastatic volume [24]. MT1-MMP proteolytic activity on the surface of cancer cells plays a central role in the invasion process. Its upregulation is associated with malignancy in several types of cancer, including breast cancer [25]. This transmembrane protease can activate the matrix metalloproteinases-2 and −9 (MMP-2 and MMP-9) [26,27], which, in coordination with them, cleave extracellular matrix proteins and thus create a passage for cancer cells [28,29]. In TNBC patients, elevated levels of MT1-MMP have been associated with cancer cell passage in blood vessels [30], and shorter overall survival [31].

Endocytic and exocytic cycles of MT1-MMP depend on Rab4A [25,32]. Rab4A is a member of the Rab GTPases family that coordinates vesicle traffic [33,34]. The importance of Rab4A in invasion and metastasis formation is supported by the progressive increase of its expression from normal tissue to primary tumor and then to metastases [35]. Rab4A expression is amplified in several tumors and particularly in invasive breast carcinoma [35,36]. The enzyme isoprenylcysteine carboxylmethyltransferase (ICMT) plays a crucial role in regulating the carboxylmethylation, proper localization, and activation of Rab4A. Inhibiting ICMT attenuates RAB4A‐mediated integrin β3 recycling, cell migration, and metastasis in a breast cancer model [35].

The RAB4A–MT1-MMP axis represents one of the best-characterized mechanistic links between Rab4A function and breast cancer metastasis. Indeed, Rab4A facilitates the trafficking of MT1-MMP to the plasma membrane, enhancing pericellular matrix degradation and invasion, processes essential for metastatic dissemination in a mouse model of breast cancer [36].

Nevertheless, it should be noted that Rab4A also plays other roles in cancer metastasis. In breast cancer cells (MDA-MB-231), Rab4A was shown to regulate tumor formation, sphere-forming ability, and epithelial-to-mesenchymal transition (EMT), driven invasion by controlling activation of the small GTPase RAC1 [37]. More recently, RAB4A was identified as an upstream regulator within a signaling cascade that relays signals sequentially through NUMB, NOTCH1, RAC1, and SOX2, thereby controlling the self-renewal capacity of multiple cancer cell types of diverse tissue origins via transcriptional regulation [38]. In addition to these signaling functions, RAB4A mediates endosomal trafficking events critical for cell migration. For instance, RAB4-dependent recycling of the integrin αvβ3 from early endosomes is necessary for efficient cell adhesion and spreading [39].

Given our previous findings that MT1-MMP contributes to the radiation-induced increase in breast cancer cell invasiveness [22], we therefore focused our present study on dissecting the role of MT1-MMP in this context. The role of Rab4A in radiation-stimulation of TNBC cell invasion *in vitro* and the development of metastases in animal model was determined. In order to get closer to the clinic, plasma samples from six TNBC patients were collected before RT and after the 4^th^ radiation fraction. The TNBC cells D2A1 (murine) and MDA-MB-231 (human) were treated with these plasmas and we determined if the downregulation of RAB4A prevents the stimulation of cancer cell invasion and metastasis development that were induced by the plasma collected during RT.

## Materials and methods

### Cell culture

The mouse breast carcinoma D2A1 cells derived from a spontaneous mammary tumour in a Balb/c mouse and were provided by Dr Ann F. Chambers (University of Western Ontario, London, ON, Canada). The human MDA-MB-231 cells were purchased from American Type Culture Collection. The cells were maintained in a 5% CO_2_ humidified incubator at 37°C in Dulbecco’s modified Eagle’s medium (DMEM) supplemented with 10% fetal bovine serum, 2 mM glutamine, 1 mM sodium pyruvate, 100 units per mL penicillin and 100 µg/mL streptomycin. All experiments were performed with mycoplasma-free cells. Cells were tested for mycoplasma by real time qPCR coupled to capillary electrophoresis at the RNomics Platform of the Université de Sherbrooke (https://rnomics.med.usherbrooke.ca/services/mycoplasma-detection).

### Downregulation of RAB4A and MT1-MMP

A short hairpin RNA (shRNA) mediated gene knockdown was performed as previously reported [22]. Lentiviral particles were produced in the human cell line 293T (5 x 10^6^) which was co-transfected using 48 µg lipofectamine with 6 µg of the plp1, plp2 and plp/VSV-G plasmids (Invitrogen) and 6 µg of pLKO.1-puro vector containing a shRNA sequence targeting either the murine RAB4A transcript TRCN0000088973, TRCN0000088974, TRCN0000088975, TRCN0000088976 and TRCN0000088977, or the human RAB4A transcript TRCN0000011217, TRCN0000231999 and TRCN0000232000 (Sigma-Aldrich). As control, the PLKO.1 non-target control DNA (code SHC016) was used. The cultured cell supernatant containing the lentivirus was collected 48 h later, filtered with a 0.45 µm membrane and kept at -80°C for further use.

The downregulation of MT1-MMP proteolytic activity by shRNA in D2A1 cells was previously reported [22]. A 70% reduction of MT1-MMP mRNA was confirmed by quantitative polymerase chain reaction (qPCR) and at the protein level by Western blot. The ability of MT1-MMP to cleave inactive proMMP-2 to active MMP-2 in D2A1 MT1-MMP knockdown cells was reduced by more than 90% compared to wild-type D2A1 cells. Nomenclature of the derived D2A1 cell lines is as follows: D2A1-WT (wild-type), D2A1 RAB4A-scramble, D2A1 KD RAB4A (RAB4A downregulated) and D2A1 KD MT1-MMP (downregulation of the Mmp14 transcript level) cells. For those derived from the MDA-BM-231 cells: MDA-MB-231-WT, MDA-MB-231 RAB4A-scramble, MDA-MB-231 KD RAB4A.

### Western blot analysis

Down regulation of RAB4A was confirmed by Western blot. Briefly, cells were plated in 100 mm plates and harvested 24h later in 600 µl lysis buffer (150 mM NaCl, 50 mM Tris-HCl, pH 8.0, 0.5% deoxycholate, 0.1% SDS, 10 mM Na_4_P_2_O_7_, 1% IGEPAL, and 5 mM EDTA) supplemented with protease inhibitors (10 µM chymostatin, 10 µM leupeptin, 9 µM antipain and 9 µM pepstatin). Sample buffer was added to each reaction before boiling tubes for 5 minutes. All reactions were analyzed by Western blot (30 µg proteins/lane) using the anti-Rab4A (Santa Cruz Biotechnology # sc-517263) and anti-actin (C4) (Santa Cruz Biotechnology) antibodies. Experiments were done 3 times and densitometry analyses were carried out using NIH Image J software.

### Analysis of the proteolytic activity of MT1-MMP

A fluorogenic peptide was used to measure the proteolytic activity of MT1-MMP on cell surface [40]. The D2A1 or MDA-MB-231 cells (3 x 10^4^ in 96-well plate) were incubated at 37°C with 15 µM of the fluorescence resonance energy transfer (FRET) peptide MMP-14 Substrate I (Sigma 444258-Calbiochem) in the reaction buffer (100 mM TRIS, 5 mM CaCl_2_, 0.01% BRIJ-35, 1 µM ZnCl_2_). The enzymatic activity was recorded according to the variation of relative fluorescent units per second (RFU/sec). The kinetics of peptide cleavage were followed for 30 min using the 96-well plate reader Synergy HT (Bio-Tek Instrument) set at λex = 340 nm and at λem = 400 nm.

### Collection of plasmas from TNBC patients

The research protocol was approved by the Research Ethics Committee, CIUSSS de l’Estrie CHUS, Quebec, Canada (protocol # MP-31-2015-930, 14–205). Six patients with pathology-confirmed TNBC status and primary tumor removed by breast-conserving surgery were enrolled. Their clinical characteristics are listed in Table 1.

**Table 1:** TNBC patients’ clinical characteristics.

	Patients					
	1	2	3	4	5	6
Age (years)	48	71	30	46	76	29
Staging	cT2 N0 M0	pT3N1aM0	T3N0M0	cT1N0M0	pT1bN0M0	cT1N0M0
Tumor grade	3	3	2	3	2	3
Distant metastasis at diagnosis	No	No	No	No	No	No
BRCA1 and BRCA2	Normal	N.D.	Normal	Normal	N.D.	Normal
Ki67	> 95%	80-90%	N.D.	N.D.	N.D.	N.D.
Neo-adjuvant chemotherapy	Yes	Yes	Yes	Yes	No	Yes
Radiotherapy	40 Gy/15 10 Gy/ 4	26 Gy/ 5 12.5 Gy/ 5	40 Gy/ 15	26 Gy/ 5 10 Gy/ 4	26 Gy/ 5	40 Gy/15 10 Gy/ 4
Radiodermatitis grade	2	1	1	1	1	1
Pembrolizumab	No	No	Yes	No	No	No

N.D.: Not determined.

A first blood sample was collected in an EDTA tube just before the first fraction of radiation (plasma before RT) and the second after the 4th fraction of radiation (plasma during RT). Plasmas were isolated by centrifugation at 1 200 g for 30 min within 40 min of collection. Aliquots of plasma were stored at −80°C.

### Cancer cell invasion assay

A volume of 100 µL of Matrigel (growth factor reduced, Corning) diluted 1/40 in cold DMEM 0.1% BSA was added on the porous membrane of cell culture insert (cellQART, Sterlitech) that was deposited in 24-well plate and incubated for 2 h at 37°C to allow the polymerization of Matrigel. The liquid layer on the polymerized Matrigel was then removed. The TNBC cells D2A1 or MDA-MB-231 and their derivatives were incubated in 0.1% BSA DMEM medium for 24 h and then added (2 x 10^4^) on the Matrigel layer, while the lower compartment of the chamber contained either 1% plasma before RT, 1% plasma during RT, 1% FBS or 0.1% BSA in DMEM media. After 24 h at 37°C, cells on the top of the porous membrane were removed with cotton swabs while those on the bottom surface were stained with 0.4% crystal violet and then counted under a microscope. The ability of plasma collected during RT to increase cancer cell invasion was reported by the number of cells that have crossed the layer of Matrigel in presence of 1% plasma collected during RT/ 1% plasma collected before RT.

### Quantification of lung metastases

The experimental protocol in mice (Ref. # 2018-1996, 013-18) was approved by the ethical committee of Université de Sherbrooke and was conformed to the regulations of the Canadian Council on Animal Care. For the welfare of the mice and to minimize their suffering and distress, monitoring and care were carried out in accordance with the Canadian Council on Animal Care recommendations. The D2A1-WT, D2A1 RAB4A-scramble, D2A1 KD RAB4A cells were transfected with the fluorescent proteins FUCCI as previously reported to detect the lung metastases in mice by optical imaging [21]. These D2A1 cells were incubated during 24 h at 37°C in DMEM medium supplemented with either 1% plasma collected before RT, 1% plasma collected during RT, or in DMEM 0.1% BSA. The cells were washed twice by centrifugation in PBS and then injected (10^5^) in the tail vein of five female Balb/c mice (Charles River) per group. The mice were monitored and weighed every 2–3 days to ensure that the development of lung metastases was not causing discomfort. Twenty-eight days later, the mice were euthanized with slow exposure of CO_2_, the lungs were collected, and the number of metastases was quantified with the epifluorescence microscope EVOS FL Auto Imaging System (Life Technologies) equipped with green (470/525 nm) or red (531/593 nm) light cubes. No mouse died before meeting the end point. Lung slices were stained by H&E and the presence of metastases were confirmed by a pathologist.

### Statistical analysis

Results are expressed as the mean ± standard error of the mean of 2–4 experiments performed in triplicate, or 5 mice per group regarding the metastasis assay. Statistical analyses were performed with PRISM v4.0 (GraphPad Software) using the Student’s *t*-test and two ways ANOVA. A value of *p* < 0.05 was considered to be statistically significant. *p < 0.05, **p < 0.005, ***p < 0.0005 and ****p < 0.00005.

## Results

### Downregulation of RAB4A expression reduces proteolytic activity of MT1-MMP

**Fig 1:**
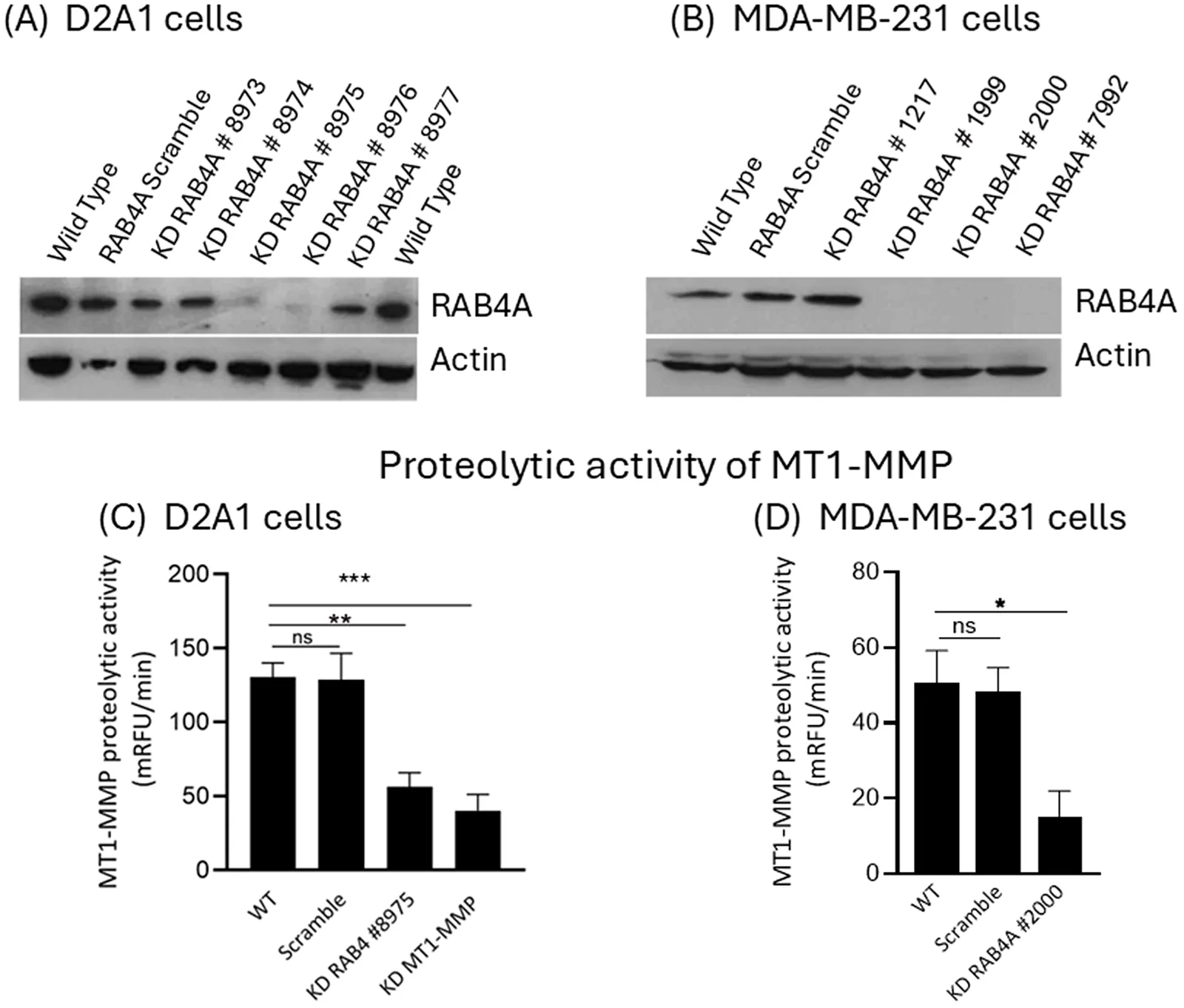
Downregulation of RAB4A reduces MT1-MMP proteolytic activity. Western blot illustrating the downregulation of RAB4A mediated by shRNA in (A) TNBC murine D2A1 cells and (B) human TNBC MDA-MB-231 cells. Downregulation of RAB4A decreased MT1-MMP proteolytic activity as determined with the fluorogenic peptide MMP-14 Substrate I. (C) A reduction by 2.3-fold was measured in the D2A1 KD RAB4A cells and (D) 3.4-fold in the MDA-MB-231 KD RAB4A cells. *p < 0.05, **p < 0.005, ***p < 0.0005.

The downregulation by shRNA of RAB4A expression in the D2A1 (murine) and MDA-MB-231 (human) cells was confirmed by Western blot analysis (Fig 1A and B). The level of Rab4A protein was more than 95% lower in the clone D2A1 KD RAB4A (shRNA sequence TRCN0000088975) and the clone MDA-MB-231 KD RAB4A (shRNA sequence TRCN0000232000), compared to their respective wild-type cells and shRNA scramble derivatives. These two clones were used for the subsequent assays.

A proteolytic assay with the fluorogenic probe MMP-14 Substrate I was performed to confirm that downregulating RAB4A reduces MT1-MMP activity at the cell surface (Fig 1C and D). As control, the proteolytic activity of MT1-MMP was measured in the D2A1 KD MT1-MMP cells. For these cells, we have previously reported a 70% reduction of MT1-MMP proteolytic activity as measured by quantitative polymerase chain reaction (qPCR) and at the protein level by Western blot [22]. As expected, downregulation of the MT1-MMP gene decreased by 3.2-fold its proteolytic activity on the cell surface compared to D2A1-WT and D2A1 RAB4A-scramble cells (*p* < 0.0005) (Fig 1C). Downregulation of RAB4A led to a 2.3-fold reduction in MT1-MMP proteolytic activity in D2A1 KD RAB4A cell (*p* < 0.005), and of 3.4-fold reduction in MDA-MB-231 KD RAB4A cells (*p* < 0.05). The shRNA scramble sequence did not significantly alter RAB4A expression or MT1-MMP proteolytic activity in the D2A1 RAB4A-scramble cells and MDA-MB-231 RAB4A-scramble cells.

### Reduction of cancer cell invasion *in vitro* by downregulating RAB4A

**Fig 2:**
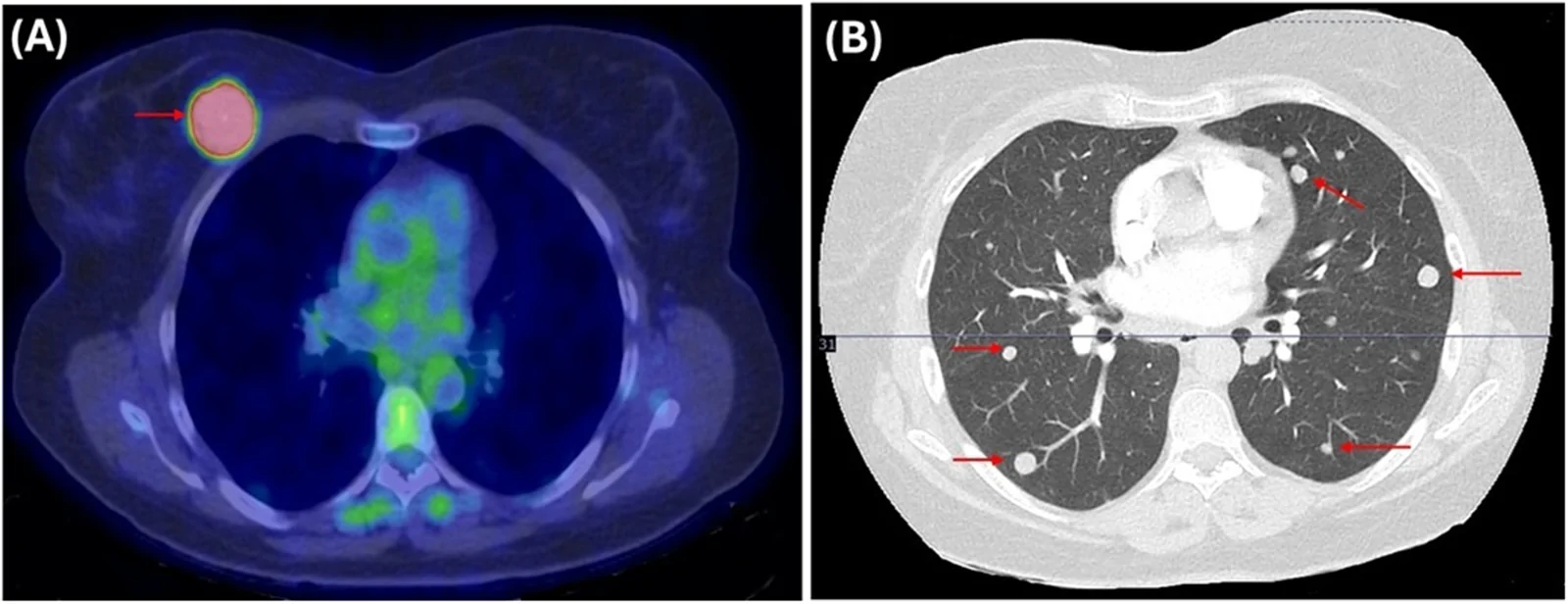
(A) Primary tumor located in a breast of TNBC patient #1 detected by positron emission tomography/ computed tomography (PET/CT) imaging scan performed with the radiotracer [F18]-fludeoxyglucose (FDG) before any treatment. (B) Lung metastases were detected six months after RT by CT angiogram imaging. Red arrows indicate the location of primary tumor and metastases.

The role of Rab4A in cancer cell invasion was first assessed with plasma collected from patient #1, who relapsed early (Fig 2).

The appropriate plasma concentration for the invasion assay was determined (S1 Fig in S1 File). The number of cancer cells crossing the Matrigel layer in the invasion chamber increased as the plasma concentration rose from 0.1% to 1%. At 10% plasma, clumps appeared randomly in the lower compartment of the invasion chambers, compromising the ability to obtain reliable results. The invasion assays were performed at a plasma concentration of 1% to obtain reproducible results.

**Fig 3:**
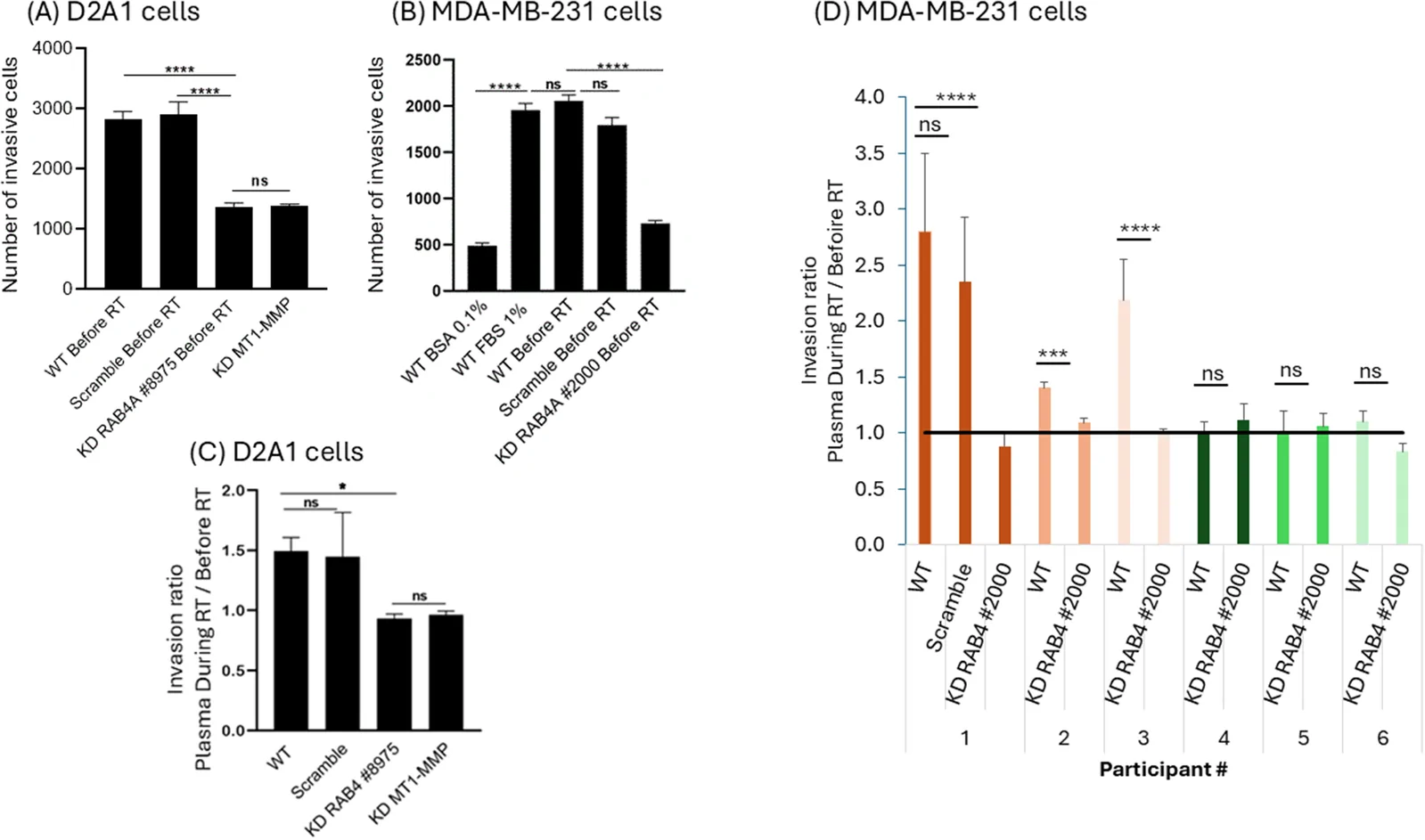
KD RAB4A blocks radiotherapy enhancement of TNBC cell invasion. The downregulation of RAB4A and MT1-MMP resulted in a decreased invasion capacity of TNBC cells when plasma collected before radiotherapy (RT) was used as an attractant (A, B). Additionally, these downregulations significantly inhibited the stimulation of invasion caused by plasma collected during RT (C, D), highlighting the relevance of targeting these pathways. D2A1 cells were incubated with plasma collected from patient #1 (C), while plasmas from patients 1 to 6 were used with the MDA-MB-231 cells (D). *p < 0.05, **p < 0.005, ***p < 0.0005 and ****p < 0.00005.

Downregulation of RAB4A or MT1-MMP in D2A1 cells reduced significantly their invasive capacity by 2.1-fold (*p* < 0.0005), compared to the wild-type counterpart, when the plasma collected before RT (patient #1) was used as attractant (Fig 3A). A similar reduction was measured with the MDA-MB-231 KD RAB4A cells that have crossed 2.9-fold less the Matrigel layer of the invasion chambers than wild-type MDA-MB-231 cells (*p* < 0.0005) (Fig 3B). As a control, no significant modification in invasion capacity was observed when these TNBC cell lines were transfected with the scrambled shRNA sequences (Fig 3A and B). In supplementary controls, 1% plasma collected before RT and 1% FBS used individually as attractant have increased similarly the invasiveness of MDA-MB-231 cells by 4.3-fold, compared to plasma-free (0.1% BSA) (Fig 3B). These results suggest that the two media had comparable chemokine levels.

### RAB4A downregulation prevents radiation-stimulation of cancer cell invasion

Plasmas collected from the TNBC patients before and during RT were added individually as attractant in the lower compartment of the invasion chambers. The results are reported according to the ratio of the number of cells having passed through the Matrigel layer with the plasma collected during the RT/ plasma collected before the RT. For D2A1 WT cells and those transfected with the shRNA scramble sequence, plasma collected during RT of patient #1 increased their invasive capacity by 1.5-fold (*p* < 0.05) (Fig 3C). A significant stimulation was also achieved with the MDA-MB-231 WT cells and their scramble clone when plasmas collected during RT from patients # 1, 2, and 3 were used as chemoattractant, with an enhancement ratio ranging from 1.4 to 2.8, compared to plasma collected before RT (patient #1, *p* = 0.002; patient #2, *p* = 3 x 10^−5^; patient #3, *p* = 0.02). Of them, two experienced a recurrence of their cancer, while the third patient had not yet completed the 5-year follow-up.

In contrast, plasma collected during RT from patients # 4, 5, and 6 who did not relapse after 5-year follow-up, did not significantly increase the invasiveness of the MDA-MB-231 cells, compared to their plasma collected before RT (Fig 3D). The responder patients (# 1, 2, and 3) and the non-responder patients (# 4, 5, and 6) were similar in terms of age, tumor grade, stage, nodal status, and the absence of detectable metastases before treatment. Additionally, they received equivalent RT doses and fractionation schedules (Table 1).

The radiation-induced stimulation of cancer cell invasion was largely blocked by downregulating RAB4A (WT vs KD RAB4A, patient #1, *p* < 0.0001; patient #2, *p* = 0.0004; patient #3, *p* < 0.0001). Indeed, the same number of D2A1 KD RAB4A and MDA-MB-231 KD RAB4A cells crossed the Matrigel layer when incubated with plasma collected before or during RT. Supporting the role of MT1-MMP, its downregulation in D2A1 KD MT1-MMP cells also largely abolish the stimulatory effect of plasma collected during RT on cancer cell invasion (Fig 3C).

### Impact of RAB4A downregulation on the development of lung metastases

The D2A1-WT, D2A1 RAB4A-scramble and D2A1 KD RAB4A cells expressing the fluorescent protein FUCCI were incubated *in vitro* in DMEM medium supplemented with either 0.1% BSA, 1% plasma collected before RT, or 1% plasma collected during RT from patient #1. Twenty-four hours later, they were washed and injected i.v. into tail of female Balb/c mice. The number of lung metastases were quantified 28 days later with the epifluorescence microscope EVOS FL Auto Imaging System.

**Fig 4:**
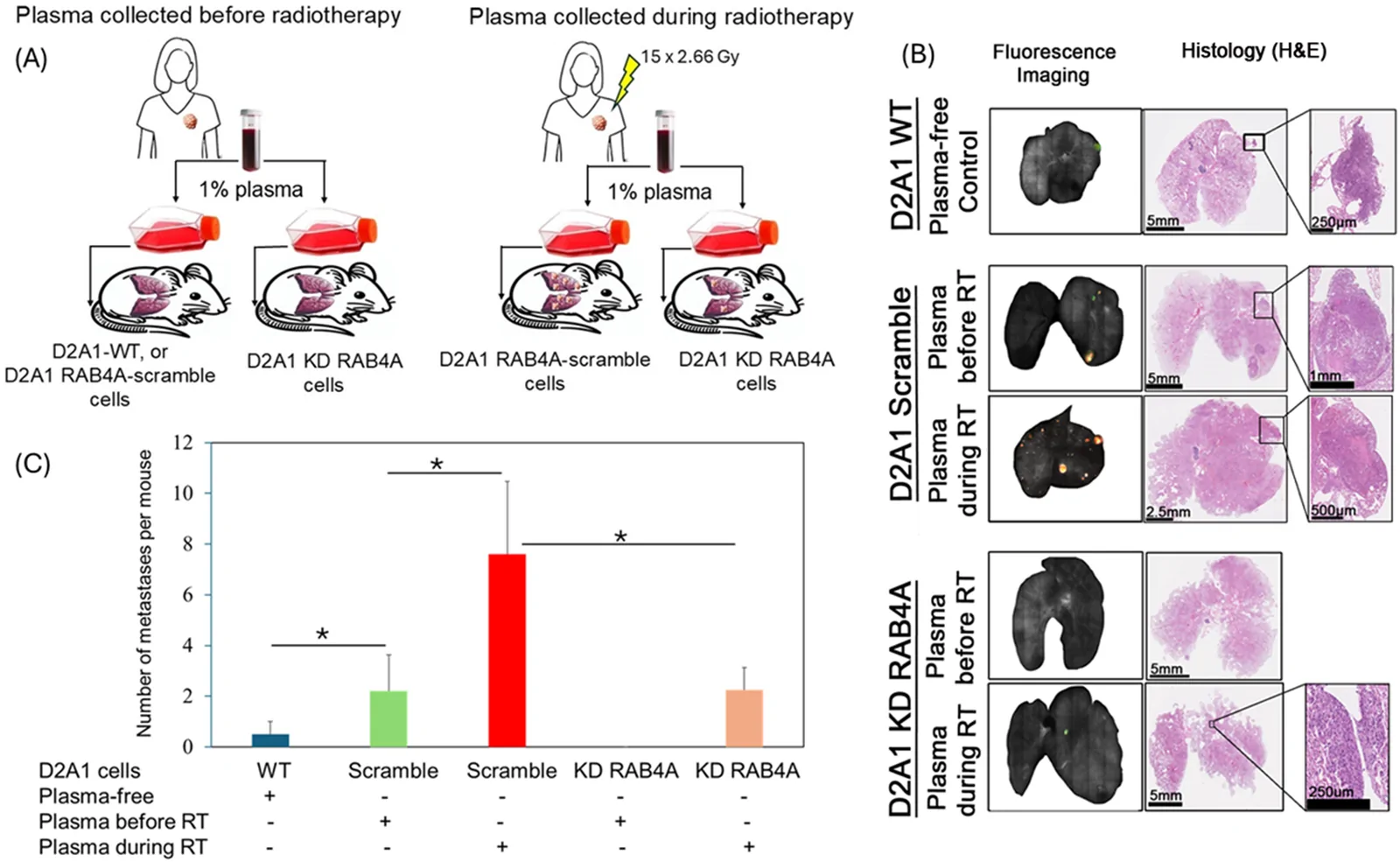
Downregulation of Rab4A prevents metastasis development induced by plasma collected from TNBC patient #1 during RT. (A) Plasma samples were collected before and during RT from TNBC participant #1 who relapsed within one year of treatment. These plasmas were then incubated with D2A1 TNBC cells and subsequently injected intravenously into Balb/c mice. (B) Lung metastases were quantified 28 days later using fluorescence imaging, and their presence was confirmed by histology. (C) Development of lung metastases by plasma collected during RT was averted by downregulating RAB4A. *p < 0.05.

Plasma collected before RT slightly increased the mean number of metastases, reaching 2.2 ± 1.4 per mouse, compared to 0.5 ± 0.5 for the control incubated with 0.1% BSA. A more significant amplification was observed with plasma collected during RT, increasing the mean number of metastases to 7.6 ± 2.9 (*p* < 0.05) (Fig 4).

RAB4A downregulation completely blocked metastasis development when D2A1 KD RAB4A cells were preincubated with plasma collected before RT. Interestingly, this protective effect was also obtained when plasma collected during RT was tested with the D2A1 RAB4A cells, resulting in a reduction of metastases to 2.3 ± 0.9, compared to 7.6 ± 2.9 when this plasma was incubated with the D2A1 RAB4A-scramble cells (*p* < 0.05).

## Discussion

The relapse rate in early-stage TNBC patients is significantly higher compared to the other breast cancer subtypes [3]. This observation highlights the need to identify new molecular targets to develop drugs capable of preventing the development of metastases which are detected after treatments. This study aims to evaluate the relevance of RAB4A as a potential target to achieve this objective.

Rab4A expression progressively increases from normal tissue to primary then to metastatic tumor [35]. Rab4A is amplified in various tumors, especially in invasive breast cancers [35,36], and its high expression correlates with poorer prognosis [35,36,41]. Gathering evidence indicates that Rab4A is required for development of metastases [35,36,41–46]. Rab4A overexpression in MDA-MB-231 cells drives tumor cell invasive migration in 3D Matrigel assays [47]. Silencing of Rab4A or expression of a dominant-negative (Rab4DN) mutant in MDA-MB-231 and MCF10.DCIS.com breast cancer cells reduced MT1-MMP recycling, extracellular matrix degradation and invasion in 3D gel matrices, and importantly, inhibited cancer cell invasiveness in mammary gland and lung tissue in immunodeficient mice [36].

The current study supports that downregulation of Rab4A reduces MT1-MMP proteolytic activity, a protease involved in cancer cell invasion. This reduction in MT1-MMP resulted in decreased invasiveness of the tested TNBC cells, and inhibited radiation-induced cancer cell invasion and development of metastasis. The development of metastases results from a multi-step process, including the invasion of cancer cells from the primary tumor into surrounding tissues, intravasation into the bloodstream, and extravasation to a distant organ where cancer cells colonize and grow [13]. A key molecular event is the cleavage of the extracellular matrix proteins by MMP, which creates a pathway for cancer cells to colonize distant organs. Their essential role in tumor progression has generated considerable interest in developing inhibitors to target them. However, convincing clinical results are still awaited because of the poor bioavailability and selectivity of MMP inhibitors [48–50]. One constraint to their clinical application could be the significant role of MMPs in fundamental physiological processes, such as promoting angiogenesis, cell migration, modulation of inflammation response and osteoclast activity [51].

MT1-MMP is also essential in numerous physiological processes [51]. However, its localization on the surface of cancer cells could offer an advantage in managing cancer progression. Over the past decade, inhibitors of MT1-MMP have been developed [52]. Although they can bind to the active sites of MT1-MMP, their overall effectiveness has remained limited [48]. The past failures in clinical trials have been mainly associated to adverse effects. Waiting for more MT1-MMP-specific inhibitors [52], we propose a novel approach which is to reduce MT1-MMP trafficking on the surface of cancer cells by inhibiting RAB4A.

To assess the potential of this new opportunity, expression of RAB4A have been downregulated by shRNA in the D2A1 and MDA-MB-231 cells. The reduction of MT1-MMP on surface of these cancer cells surface was confirmed by a significantly decreased of its proteolytic activity, resulting in a marked loss of invasion capacity *in vitro* for the D2A1 and MDA-MB-231 cells.

Although this approach is promising, RAB4A inhibition cannot be performed in disease-free individuals to prevent the development of metastases because MT1-MMP is involved in multiple physiological processes. A RAB4A inhibitor should therefore be administered to eliminate metastases or to prevent their development in early-stage TNBC patients who are initially diagnosed as free of metastases.

Emerging evidence indicates that the inflammatory response triggered by RT may promote critical pathways in certain patients, such as increased cancer cell invasion, a higher number of circulating tumor cells, and the development of new metastases [21,53–55]. Ongoing clinical trials will provide further results to help understand the association between RT and the increased risk of developing metastases for certain patient groups. In the meantime, it is relevant to evaluate whether an RAB4A inhibitor could block RT stimulation of metastasis development.

In our study, TNBC patients were recruited to determine whether downregulating RAB4A might block RT stimulation of some critical steps in cancer progression. Plasma samples were collected before and after the 4^th^ fraction of RT in patients with an early-stage TNBC. At diagnosis, the absence of metastases was confirmed by positron emission tomography (PET) or CT scan. Plasma samples collected during RT from three of these patients increased the invasiveness of TNBC cells. Of them, two experienced a recurrence of their cancer, while the third patient had not yet completed the 5-year follow-up. This adverse effect of RT was mitigated by downregulating RAB4A. In contrast, in the three patients who did not relapse after the 5-year follow-up, plasma collected during RT did not significantly increase the invasiveness of the MDA-MB-231 cells, compared to their plasma collected before RT. For these patients, the decrease in RAB4A expression did not result in a significant difference in the invasiveness of cancer cells when incubated with plasma collected before or during RT.

Taken together, RAB4A downregulation appears to inhibit both the baseline and RT-stimulated invasion capacities of D2A1 and MDA-MB-231 cells. To investigate this hypothesis, additional assays could determine whether plasma samples collected during RT from responder patients increase the MT1-MMP expression and its proteolytic activity compared with plasma samples collected before RT. These results could then be compared with those obtained from plasma samples collected from non-responder patients, in which plasma collected during RT should not increase MT1-MMP proteolytic activity. Additionally, pre-incubating D2A1 cells *in vitro* with plasma collected during RT from patient #1, who relapsed early, and then injected intravenously into the tail vein of mice significantly enhanced the development of lung metastases. This enhancement was not observed with plasma collected from the same patient prior to RT. The TNBC patient #1 appears to belong to a subgroup where early recurrence is linked to an increased ability of cancer cells to invade and metastasize after RT. Therefore, she was a suitable candidate to assess whether downregulating RAB4A could help to block these side effects of RT. Our preclinical data clearly show the downregulating RAB4A markedly reduces RT stimulation of cancer cell invasion and metastasis development. These results were expected since similar protective effects were reported in preclinical models when MT1-MMP was downregulated [22,30]. These results suggest that radiation may have increased the level of inflammatory cytokines, such as TNF-α, in plasma collected during RT from patient #1. This cytokine can activate the NFκB signaling pathway, thereby upregulating MT1-MMP gene expression [56].

Our study has some limitations. A larger number of TNBC patients, with and without recurrence, would strengthen the association between plasma samples collected during RT and the increase in cancer cell invasiveness induced by this treatment. Additionally, our murine model only measured the final stage of metastasis development, where circulating cancer cells reach the lungs and infiltrate and grow in that organ. However, the role of RT in the entire process has been demonstrated by irradiating a TNBC tumor implanted in the mammary gland of mice, which increases the number of circulating tumor cells and lung metastases [21]. Our study demonstrated the role of Rab4A in maintaining MT1-MMP proteolytic activity, but its association with other proteases has not been investigated. Validation of the role of RAB4A in RT-mediated cancer cell invasion could have been strengthened by using a second shRNA or a rescue experiment. To further strengthen the role of RAB4A as a regulator of cell-surface trafficking of MT1-MMP, a flow cytometry assay with appropriate antibodies could have been performed.

## Conclusions

Reducing the relapse rate occurring within the first 3 years following treatment for early-stage TNBC patients still represents a significant challenge in improving the management of these patients. Since RT remains an essential component in the treatment options for TNBC, the next step is to develop a complementary treatment that will reduce the incidence of recurrence that could be associated with RT. Among the targets that deserve to be exploited, inhibition of the ability of RAB4A to recycle MT1-MMP on the surface of cancer cells could prove promising. Along with developing this inhibitor, it will be necessary to determine the period of its administration before, during and after the RT plan to ensure that patients receive benefits while maintaining good tolerance. On the other hand, although cleavage of extracellular matrix proteins is a key step in the invasion of cancer cells and development of metastases, the probability of relapse depends on other factors, such as the ability of the antitumor immune response to prevent metastasis development. Therefore, targeting RAB4A to reduce cancer cell seeding to distant organs may need to be combined with a complementary therapy, for example, one that promotes an antitumor immune response. In summary, our preliminary results suggest that RT may promote cancer cell invasion and the formation of metastases, which could be inhibited by targeting Rab4A, responsible for regulating the turnover of MT1-MMP on the surface of cancer cells

## Supporting information

S1 File Raw data. (DOCX)

S2 File Graphical abstract. (TIF)
