# Supplementary material for: Role of Rab4A in the enhancement of cancer cell invasion induced by plasma samples collected during radiotherapy in patients with triple-negative breast cancer
Source: PLoS One. 2026 Sep 15;21(9):e0329717. doi: 10.1371/journal.pone.0329717 (PMC13577393; doi:10.1371/journal.pone.0329717)
Supplement: Supplementary file 1 [file pone.0329717.s001.docx]

**Role of Rab4A in the enhancement of cancer cell invasion induced by plasma samples collected during radiotherapy in patients with triple-negative breast cancer**

Benoit Paquette, Hélène Therriault, Isabelle Gauthier, Sawyna Provencher, Jean-Marc Bourque, Sahar Naasri, Ayman Oweida, Sameh Geha, Pascale Labrecque and Jean-Luc Parent

**Raw data**

Figure 1C

| MT1-MMP Proteolytic activity (mRFU / min) | | | | | |
| --- | --- | --- | --- | --- | --- |
| D2A1 cells | | | | |  |
|  | WT | Scramble | KD RAB4 #8975 | KD MT1-MMP |  |
|  | 137.0 | 167.7 | 65.9 | 23.6 |  |
|  | 111.7 | 99.6 | 46.8 | 14.9 |  |
|  | 142.9 | 97.6 | 34.4 | 30.3 |  |
|  |  | 150.4 | 59.9 | 65.3 |  |
|  |  |  | 74.2 | 67.6 |  |
| Mean | 130.5 | 128.8 | 56.2 | 40.3 |  |
| SEM | 9.6 | 17.8 | 7.0 | 10.9 |  |
| P value VS WT |  | 0.94 | 0.0014 | 0.0005 |  |

Figure 1D

| MT1-MMP Proteolytic activity (mRFU / min) | | | |
| --- | --- | --- | --- |
| MDA-MB-231 cells | | | |
|  | WT | Scramble | KD RAB4 #2000 |
|  | 59.3 | 34.7 | 0.0 |
|  | 65.4 | 58.7 | 3.4 |
|  | 25.8 | 71.7 | 16.9 |
|  | 32.7 | 51.6 | 0.0 |
|  | 90.7 | 31.4 | 39.1 |
|  | 42.4 | 42.6 | 30.8 |
|  | 38.6 |  |  |
| Mean | 50.7 | 48.4 | 15.0 |
| SEM | 8.5 | 6.2 | 6.9 |
| P value VS WT |  | 0.97 | 0.01 |

Figure 3A

| Number of invasive D2A1 cells | | | | |  | |
| --- | --- | --- | --- | --- | --- | --- |
|  | WT Before RT | Scramble  Before RT | KD RAB4A #8975 Before RT | KD MT1-MMP | |  |
|  | 2987 | 3286 | 1420 | 1366 | |  |
|  | 3027 | 3330 | 1447 | 1384 | |  |
|  | 3052 | 3357 | 1265 | 1396 | |  |
|  | 2772 | 3049 | 1349 | 1267 | |  |
|  | *3205* | 3526 | 1038 | 1465 | |  |
|  | 2400 | 2640 | 1745 | 1456 | |  |
|  | 2406 |  |  |  | |  |
| Mean | 2836 | 3198 | 1377 | 1389 | |  |
| SEM | 121.6 | 128 | 94.9 | 29.3 | |  |
| P value VS WT |  | 0.51 | <0.0001 | <0.0001 | |  |
| P value VS KD RAB4 |  |  |  | 0.9101 | |  |

Figure 3B

|  |  |  | Number of invasive MDA-MB-231 cells | | |  |
| --- | --- | --- | --- | --- | --- | --- |
|  |  | *WT BSA 0.1%* | WT FBS 1% | WT Before RT | Scramble Before RT | KD RAB4A #2000 Before RT |
|  |  | *421* | 1924 | 2163 | 1795 | 730 |
|  |  | 455 | 2222 | 2142 | 1778 | 723 |
|  |  | 568 | 2133 | 1973 | 1638 | 666 |
|  |  | 569 | 2141 | 1972 | 1971 | 801 |
|  |  | 583 | 2249 | 1826 | 2059 | 837 |
|  |  | 381 | 1887 | 1800 | 1516 | 616 |
|  |  | 421 | 1671 | 2198 |  |  |
|  |  |  | 1848 | 2281 |  |  |
|  |  |  | 1454 | 2130 |  |  |
|  |  |  | 1888 | 1771 |  |  |
|  |  |  | 2092 | 2378 |  |  |
|  | Mean | 485 | 1955 | 2058 | 1793 | 729 |
|  | SEM | 32.17 | 74 | 61 | 82 | 33 |
| P value VS WT | |  |  | 0.3519 | 0.1244 | <0.0001 |
| P value VS 0.1% BSA | |  | <0.0001 |  |  |  |

Figure 3C

|  | | D2A1 Cell invasion: During RT / Before RT | | | | | |
| --- | --- | --- | --- | --- | --- | --- | --- |
|  |  |  | WT | Scramble | KD RAB4 #8975 | KD MT1-MMP |  |
|  |  |  | 1.28 | 0.71 | 1.08 | 0.99 |  |
|  |  |  | 1.53 | 0.98 | 0.96 | 0.97 |  |
|  |  |  | 1.67 | 2.31 | 0.9 | 0.93 |  |
|  |  |  |  | 1.79 | 0.94 |  |  |
|  |  |  |  |  | 0.92 |  |  |
|  |  |  |  |  | 0.8 |  |  |
|  |  | Mean | 1.49 | 1.45 | 0.93 | 0.96 |  |
|  |  | SEM | 0.11 | 0.37 | 0.04 | 0.02 |  |
|  | | P value VS WT |  | 0.92 | 0.01 | 0.02 |  |
|  | | P value VS KD RAB4 |  |  |  | 0.61 |  |

Figure 3D

|  | Patient #1 | | Invasion ratio: Plasma during RT / Before RT | | | | |
| --- | --- | --- | --- | --- | --- | --- | --- |
|  |  | | WT | | Scramble | KD RAB4A #2000 | |
|  |  | | 3.42 | | 2.77 | 1.10 | |
|  |  | | 2.92 | | 2.31 | 0.63 | |
|  |  | | 2.06 | | 1.79 | 1.03 | |
|  |  | | 2.63 | |  | 0.80 | |
|  |  | | 2.89 | |  | 0.74 | |
|  |  | | 2.80 | |  | 1.10 | |
|  | Mean | | 2.79 | | 2.29 | 0.90 | |
|  | SEM | | 0.18 | | 0.38 | 0.08 | |
|  | P value VS WT | |  | | 0.11 | <0.0001 | |
|  |  | |  | |  |  | |
|  |  | |  | |  |  | |
|  |  | |  | |  |  | |
|  |  | |  | |  |  | |
|  |  | |  | |  |  | |
|  |  | |  | |  |  | |
|  |  | |  | |  |  | |
|  |  | |  | |  |  | |
|  |  | |  | |  |  | |
|  |  | |  | |  |  | |
|  |  | |  | |  |  | |
|  |  | |  | |  |  | |
| Patient #2 | | Invasion ratio: Plasma during RT / Before RT | | | | |  |
|  | | WT | | KD RAB4A #2000 | | |  |
|  | | 1.25 | | 1.19 | | |  |
|  | | 1.25 | | 1.08 | | |  |
|  | | 1.28 | | 1.09 | | |  |
|  | | 1.66 | | 0.99 | | |  |
|  | | 1.53 | |  | | |  |
|  | | 1.35 | |  | | |  |
|  | | 1.48 | |  | | |  |
|  | | 1.4 | |  | | |  |
| Mean | | 1.4 | | 1.09 | | |  |
| SEM | | 0.05 | | 0.04 | | |  |
| P value vs WT | |  | | 0.0004 | | |  |

| Patient #3 | Invasion ratio: Plasma during RT / Before RT | |
| --- | --- | --- |
|  | WT | KD RAB4A #2000 |
|  | 2.05 | 1.07 |
|  | 2.34 | 1.04 |
|  | 1.80 | 0.83 |
|  | 2.26 | 0.76 |
|  | 2.47 | 1.09 |
| Mean | 2.19 | 0.96 |
| SEM | 0.12 | 0.07 |
| P value VS WT |  | <0.0001 |

| Patient #4 | Invasion ratio: Plasma during RT / Before RT | |
| --- | --- | --- |
|  | WT | KD RAB4A #2000 |
|  | 0.90 | 0.83 |
|  | 1.20 | 1.50 |
|  | 1.30 | 1.56 |
|  | 1.30 | 0.86 |
|  |  | 1.08 |
|  |  | 0.86 |
| Mean | 1.18 | 1.12 |
| SEM | 0.09 | 0.14 |
| P value vs WT |  | 0.36 |

| Patient #5 | Invasion ratio: Plasma during RT / Before RT | |
| --- | --- | --- |
|  | WT | KD RAB4A #2000 |
|  | 0.80 | 1.39 |
|  | 1.01 | 0.60 |
|  | 1.32 | 0.95 |
|  |  | 1.12 |
|  |  | 1.23 |
|  |  | 1.07 |
| Mean | 1.04 | 1.06 |
| SEM | 0.15 | 0.11 |
| P value VS WT |  | 0.47 |

| Patient #6 | Invasion ratio: Plasma during RT / Before RT | |
| --- | --- | --- |
|  | WT | KD RAB4A #2000 |
|  | 0.90 | 0.74 |
|  | 1.13 | 0.82 |
|  | 1.25 | 0.73 |
|  | 0.91 | 1.14 |
|  |  | 0.74 |
| Mean | 1.05 | 0.83 |
| SEM | 0.09 | 0.08 |
| P value VS WT |  | 0.06 |

Figure 4C

|  | Number of lung metastases per mouse | | | | |
| --- | --- | --- | --- | --- | --- |
|  | A | B | C | D | E |
|  | D2A1 cells WT: Plasma-free | D2A1 cells Scramble: Plasma before RT | D2A1 cells Scramble: Plasma during RT | D2A1 cells KD RAB4A #8975: Plasma before RT | D2A1 cells KD RAB4A #8975: Plasma during RT |
|  |  |  |  |  |  |
|  | 1 | 3 | 3 | 0 | 1 |
|  | 0 | 0 | 9 | 0 | 2 |
|  | 1 | 2 | 13 | 0 | 4 |
|  | 0 | 1 | 8 | 0 | 2 |
|  | 0 | 5 | 5 | 0 |  |
| Mean | 0.4 | 2.2 | 7.6 | 0.0 | 2.3 |
| SEM | 0.29 | 0.86 | 1.72 | 0.00 | 0.88 |
| P value VS A |  | 0.04 |  |  |  |
| P value VS B |  |  | 0.023 |  |  |
| P value VS C |  |  |  |  | 0.034 |

Figure S1

Number of invasive MDA-MB-231 cells

| % PLASMA | # CELLS | MEAN | SEM | P VALUE |
| --- | --- | --- | --- | --- |
|  | 615 |  |  |  |
| 0.1% plasma-Before RT | 832 | 683 | 75 |  |
|  | 602 |  |  | 0.01 |
|  | 1178 |  |  |  |
| 0.1% plasma-During RT | 1212 | 1206 | 30 |  |
|  | 1227 |  |  |  |
|  | 2141 |  |  |  |
| 1% plasma-Before RT | 2124 | 2021 | 44 |  |
|  | 1756 |  |  |  |
|  | 2062 |  |  | <0.0001 |
|  | 3452 |  |  |  |
| 1% plasma-During RT | 3444 | 3571 | 107 |  |
|  | 3500 |  |  |  |
|  | 3889 |  |  |  |
|  | 2026 |  |  |  |
| 10% plasma-Before RT | 2458 | 2268 | 127 |  |
|  | 2319 |  |  | 0.31 |
|  | 1954 |  |  |  |
| 10% plasma-During RT | 2936 | 2922 | 555 |  |
|  | 3876 |  |  |  |
